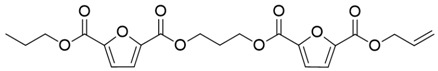# Thermal Decomposition Kinetics and Mechanism of In-Situ Prepared Bio-Based Poly(propylene 2,5-furan dicarboxylate)/Graphene Nanocomposites

**DOI:** 10.3390/molecules24091717

**Published:** 2019-05-02

**Authors:** Zoi Terzopoulou, Evangelia Tarani, Nejib Kasmi, Lazaros Papadopoulos, Konstantinos Chrissafis, Dimitrios G. Papageorgiou, George Z. Papageorgiou, Dimitrios N. Bikiaris

**Affiliations:** 1 Laboratory of Polymer Chemistry and Technology, Department of Chemistry, Aristotle University of Thessaloniki, GR54124 Thessaloniki, Greece; terzoe@gmail.com (Z.T.); nejibkasmi@gmail.com (N.K.); lazaros.geo.papadopoulos@gmail.com (L.P.); 2 Solid State Physics Department, School of Physics, Aristotle University of Thessaloniki, GR54124 Thessaloniki, Greece; etarani@physics.auth.gr; 3 School of Materials and National Graphene Institute, University of Manchester, Oxford Road, Manchester M13 9PL, UK; dimitrios.papageorgiou@manchester.ac.uk; 4 Chemistry Department, University of Ioannina, P.O. Box 1186, 45110 Ioannina, Greece; gzpap@uoi.gr

**Keywords:** poly(propylene 2,5 furandicarboxylate), graphene nanoplatelets, nanocomposites, bio-based polymers, thermal stability, decomposition mechanism

## Abstract

Bio-based polyesters are a new class of materials that are expected to replace their fossil-based homologues in the near future. In this work, poly(propylene 2,5-furandicarboxylate) (PPF) nanocomposites with graphene nanoplatelets were prepared via the in-situ melt polycondensation method. The chemical structure of the resulting polymers was confirmed by ^1^H-NMR spectroscopy. Thermal stability, decomposition kinetics and the decomposition mechanism of the PPF nanocomposites were studied in detail. According to thermogravimetric analysis results, graphene nanoplatelets did nοt affect the thermal stability of PPF at levels of 0.5, 1.0 and 2.5 wt.%, but caused a slight increase in the activation energy values. Pyrolysis combined with gas chromatography and mass spectroscopy revealed that the decomposition mechanism of the polymer was not altered by the presence of graphene nanoplatelets but the extent of secondary homolytic degradation reactions was increased.

## 1. Introduction

In recent years, polymers have become a necessary part of modern life [1]. From clothes to housing, transportation to medicine and electronics, polymeric materials promote, foster and enable a sustainable society [2]. Environmental concerns have arisen though, mainly because the vast majority of these polymers is produced from non-renewable resources [3,4], so a growing interest towards the preparation of materials and chemicals from renewable resources is observed [2,5,6,7,8]. Economic reasons, such as the fluctuation of crude oil prices since fossil fuel resources have been diminishing, along with environmental ones, such as the limited biodegradability and the significant greenhouse gas emissions from the production of fossil-based materials, have shifted the attention towards bio-based raw materials and polymers [6,9,10,11]. For renewable polymers to enter the marketplace, they must outperform the traditional ones both in price and in performance. To accomplish this, new routes for biomass conversion and monomer generation must be used, to discover new, high performance materials [1]. Renewable raw materials such as cellulose, lignin, proteins, starch and vegetable oils have been intensively explored in order to develop a sustainable bio-based economy [12,13] and their use has been promoted legislatively, both in U.S.A. and Europe [6,14]. In parallel, research towards discovering new materials is constant, as shown by numerous papers where plants [15] or bacteria [16] have been used to produce materials suitable for applications as waste water treatment and drug delivery.

One of the most important biomass derived monomers is 2,5 furandicarboxylic acid (FDCA). FDCA can be formed by oxidative dehydration of glucose or by oxidation of 5-hydroxy-methylfurfural, and is considered a bio-derived homologue of terephthalic acid (TPA) [17,18], a monomer widely used for the production of commodity plastics such as poly(ethylene terephthalate) (PET), poly(propylene terephthalate) (PPT) and poly(butylene terephthalate) (PBT) that find many applications in today’s society. In fact, FDCA is a monomer of such importance, that it has been included in the US Department of Energy list of top priority bio-based chemicals, which was published in 2004 [17]. Since that, polyesters containing furan moieties in their structure have been widely investigated, bearing promising results compared to their fossil-based counterparts [19,20,21,22,23,24]. The combination of FDCA with bio-based diols leads to fully bio-based polyesters that are already being produced on an industrial scale. Among them, poly(ethylene 2,5 furandicarboxylate) (PEF) has attracted the greatest interest, as it considered a viable alternative to PET for packaging applications, thus many parameters of its synthesis have been examined thoroughly in recent years [25,26,27,28].

Aside from PEF, many polyesters based on FDCA have been synthesized using various diols [21,29,30,31], one of them being 1,3-propanediol (PDO). Polyesters derived from PDO were not studied till recently, due to unavailability of the specific monomer in large quantities [32]. With the development of new processes of production though, the situation has changed and there has been increased interest towards this family of polyesters [33,34]. Poly(propylene terephthalate) (PPT) was the first polymer of this category that was available in the market [35]. Its applications were mainly the production of fibers, hence the odd number of methylene groups in the diol’s structure provide better resilience and stress recovery properties compared with other terephthalic homologues from diols with an even number of methylene groups [36]. Poly(propylene furanoate) (PPF) is the bio-based counterpart of PPT. Industrially, PPF is a polyester that is used in packaging applications like multilayered materials, since it has good gas barrier properties [37,38].

The necessity to improve the produced materials has led to the use of various types of fillers as means to enhance the final product’s performance. While traditional composite materials contain a big quantity of fillers bound to the polymer matrix, in nanocomposites small quantities of fillers can result in significant changes of the polymer properties, due to the enormous surface area per unit volume of the nanofillers, among other factors. Carbon nanofillers especially, ones like carbon nanotubes, graphene, and graphene oxide have been proposed as next-generation multifunctional nanofillers for the improvement of the thermal, electrical and mechanical properties of diverse matrices [39,40]. In recent publications, several types of carbon nanofillers were used enhance the properties of polymeric matrices. For example, carbon black was introduced to polyurethane polymers and it resulted in improved mechanical properties, while also increasing the wear resistance of the resulting materials [41]. The mechanical properties of polymeric materials are also enhanced by the addition of carbon fibers, especially resins. Due to their prominent strength-weight and stiffness-weight ratios, carbon fibers are excellent reinforcements for resin composites [42], and drawbacks such as poor interfacial interactions with the polymer matrix can be overcome by surface modification, resulting in materials with improved mechanical properties [43,44]. The addition of carbon nanofillers also results in materials with improved electrical properties, as shown in recent papers. For instance, graphene oxide was used to create supercapacitors that display excellent cycle stability even after 500 cycles [45]. Also, graphene sheets were found to enhance the conductivity of carbon-sulfur membranes and increasing their long term cycling stability [46]. Graphene, one of the more interesting substances in the fields of materials science, physics, chemistry, and nanotechnology, is a free-standing 2D crystal with one-atom thickness [47]. As an allotrope of carbon comprises layers of six-atom rings in a honeycombed network and can be wrapped to generate 0D fullerenes, rolled up to form 1D carbon nanotubes, and stacked to produce 3D graphite [48]. More recently, developments have been made towards the preparation of thinner forms of graphite, known as graphene nanoplatelets (GNPs). All types of graphitic material are covered by the definition of GNPs, from 100 nm thick platelets down to single layer graphene [49]. It is, however, the availability of single- or few-layer graphene that has caused the most excitement in recent times [50]. The addition of GNPs to polymers has been found to lead to substantial improvements in the thermal, mechanical and electrical properties at lower loadings than are needed with expanded graphite because of high intrinsic thermal conductivity, large specific surface area, and high two-dimensional sheet geometry [51,52,53,54,55,56,57,58].

Different types of fillers have been used for the improvement of the properties of furan-based polyesters, including nanoclays [23,57], nanocellulose [59,60,61], nanosized silica and titanium dioxide [28], graphene [62], graphene oxide and multi wall carbon nanotubes [63]. Recently, Paszkiewicz et al. [62] prepared PPF nanocomposites with low concentrations (0.1 and 0.3 wt.%) of few-layer graphene, and found that the addition of the filler did not change the glass transition temperature or the melting behavior of the polymer, but resulted in inhibition of the transport of oxygen molecules into and through the material. However, there is no information published about the effect of GNPs on the thermal degradation behavior and decomposition mechanism of PPF nanocomposites in the literature. Thermal stability of polymers and their nanocomposites is a crucial parameter that affects their thermal processing and their final applications.

In this work, neat PPF and PPF/GNP nanocomposites containing 0.5, 1, and 2.5 wt.% GNPs were synthesized in-situ by melt polycondensation. Then, the chemical structure of the resulting polymers was studied by ^1^H-NMR spectroscopy. The effect of GNPs content on the thermal stability and thermal degradation of these polyesters along with a decomposition kinetics study was performed using thermogravimetric analysis (TGA), in order to reveal similarities and differences on their decomposition mechanism. Furthermore, pyrolysis-gas chromatography/mass spectroscopy (Py-GC/MS) was employed on the PPF/GNP nanocomposites in order to identify the individual fragments from each sample and obtain structural information concerning the decomposition mechanism.

## 2. Results and Discussion

### 2.1. Synthesis and Molecular Characterization

**Scheme 1:**
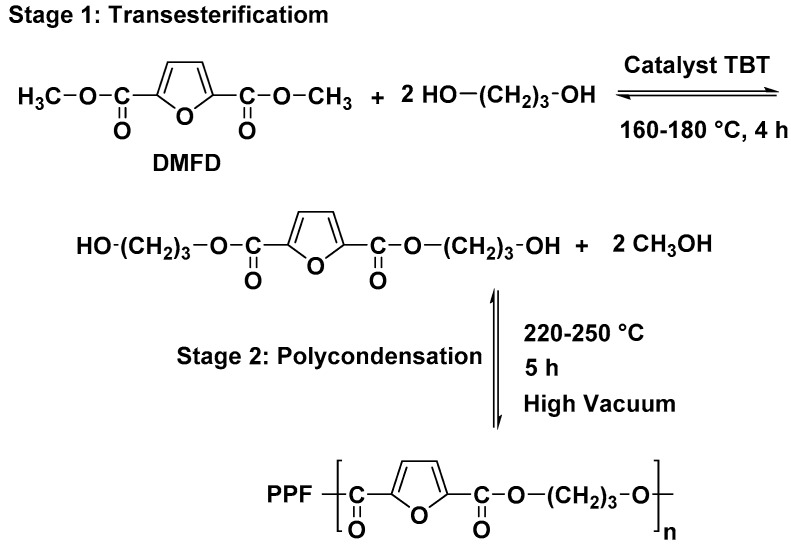
Synthesis of PPF.

The PPF nanocomposites were synthesized via the two-step melt polycondensation method. The reaction procedure is presented in Scheme 1. The obtained nanocomposites were black solids.

**Figure 1:**
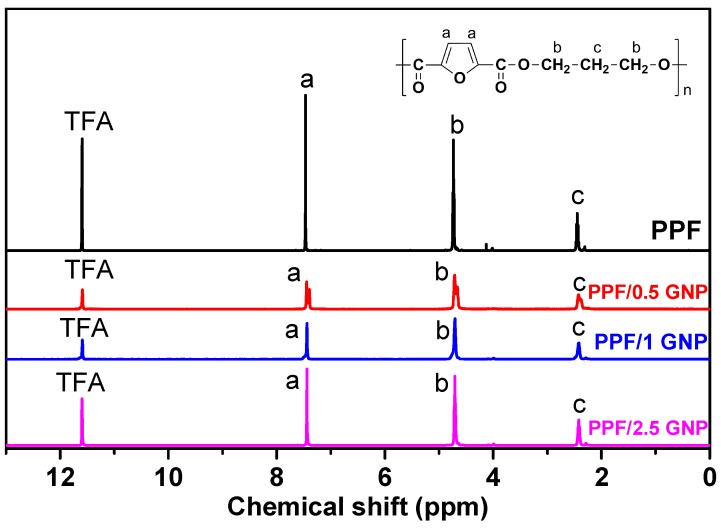
PPF structure, ^1^H-NMR spectra and peak assignments of PPF and PPF/GNP nanocomposites.

The intrinsic viscosity values of PPF and PPF/GNP nanocomposites with 0.5, 1, 2.5 wt.% GNPs were 0.62, 0.51, 0.5 and 0.57, respectively. The structure of the prepared materials was verified by ^1^H-NMR spectroscopy, presented in Figure 1, along with the peak assignments. In the spectrum of neat PPF, the propylene glycol protons appear at lower values, at 4.75 ppm for the protons near the oxygen atoms (b) which are the most deprotected, and at 2.44 ppm for the (c) protons. As for the furan ring protons (a), they are the most deprotected, due to the π electron system of the ring and the carbonyl groups, and they appear at 7.45 ppm. The above results are in accordance with our previous work [23]. The same peaks were recorded for the nanocomposites thus confirming that the addition of GNPs had no effect on the molecular structure of the prepared polyesters.

**Figure 2:**
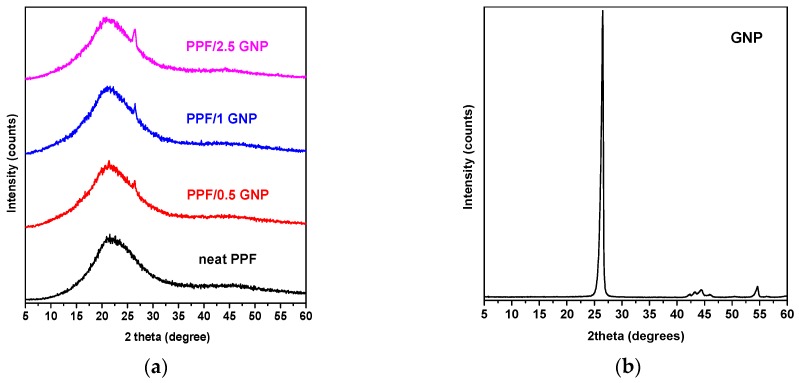
X-ray diffraction patterns of (**a**) PPF/GNP nanocomposites; (**b**) GNP.

The crystalline structure of the materials was assessed with WAXD analysis. As seen in the diffractograms of Figure 2, PPF and PPF/GNP nanocomposites were found to be amorphous, as they present a broad peak at 2θ = 22°. In the second diffractogram (Figure 2b) it is observed that the GNPs present a peak at 2θ = 26.5°, with d = 3.35 Å. In the nanocomposite materials, the same peak can be seen in all three polymers, and its intensity is increasing by increasing the loading of the material with the nanofiller, evidence of an intercalated polymer/GNPs structure. Similar results were shown in other studies examining graphene nanocomposites [64,65].

### 2.2. Thermal Stability of PPF/GNP Nanocomposites

**Figure 3:**
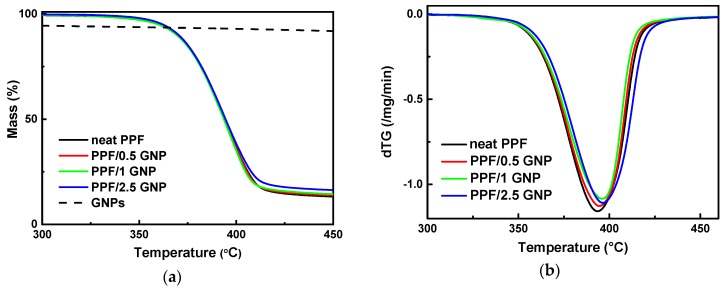
(**a**) TGA thermograms; (**b**) dTG curves of PPF/GNP nanocomposites with different weight fractions of GNPs recorded with the heating rate of 10 °C/min under a nitrogen atmosphere.

Thermogravimetric analysis (TGA) was performed with the objective of determining the thermal stability, as well as the influence of GNP content (0.5–2.5 wt.%) on the thermal properties of PPF. The TGA thermograms and derivative mass loss (dTG) curves of PPF/GNP nanocomposites with different filler content at a heating rate of 10 °C/min under nitrogen atmosphere are shown in Figure 3. In Figure 3a, the TGA curve of the GNPs is also presented (dashed curve) for comparative purposes. Analyzing the TGA results, it can be deduced that no remarkable mass loss has occurred until 325 °C, proving the excellent thermal stability of PPF-based materials. Mass loss curves of all studied samples are seemingly almost identical up to ~400 °C, consisting of a one-step procedure and obtaining the same curve shape. Above 400 °C the neat PPF and PPF/GNP nanocomposites decomposed. The residual amount of the nanocomposites increased with increasing the content of GNPs, indicating that thermal decomposition of PPF is retarded in the nanocomposites, due to the decomposition of GNPs that have initial degradation temperature above 600 °C (TGA thermograms of GNPs- dashed curve). From the dTG curves, it can be concluded that the degradation is carried out as a one-step process for all the studied samples as only one peak is observed. Concerning the effect of filler’s content on the thermal properties of PPF, GNPs seem to have little effect on the temperature that the maximum decomposition rate takes place T_d,max_, Table 1. A very small enhancement in thermal stability at the initial stage of degradation, of nearly 3 °C, and 6 °C appeared for PPF/0.5 GNP, and PPF/2.5GNP composites, respectively, using the temperature at 2% weight loss as a comparison point. Thermal stability is very important for polymeric materials as it is often the limiting factor both in processing and in end-use applications.

### 2.3. Thermal Degradation Mechanism of Neat PPF and PPF/2.5GNP Nanocomposite

**Figure 4:**
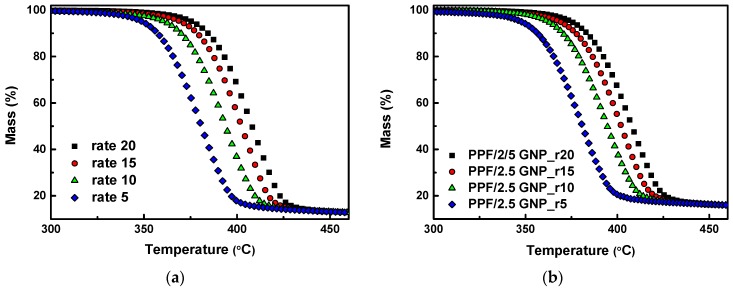
Mass (%) curves of (**a**) neat PPF; (**b**) PPF/2.5 GNP nanocomposite at heating rates of 5, 10, 15, and 20 °C/min under nitrogen.

As was already concluded from the mass loss curves (TGA) and dTG of the PPF/GNP nanocomposites with 0.5, 1 and 2.5 wt.% of GNPs that the difference between them is very small. In order to analyze more thoroughly the degradation mechanism of PPF and PPF/2.5 GNP composite, the composite which presented the highest thermal stability enhancement, kinetic parameters (activation energy *E*, pre-exponential factor *A*) and the conversion function f(α) must be evaluated. So, neat PPF and PPF/2.5GNP nanocomposite was studied using a model fitting method. The relationship between kinetic parameters and extent of conversion (*α*) for neat PPF and PPF/2.5 GNP nanocomposite can be found using the mass curves recorded at four heating rates of 5, 10, 15, and 20 °C/min under nitrogen (Figure 4).

**Figure 5:**
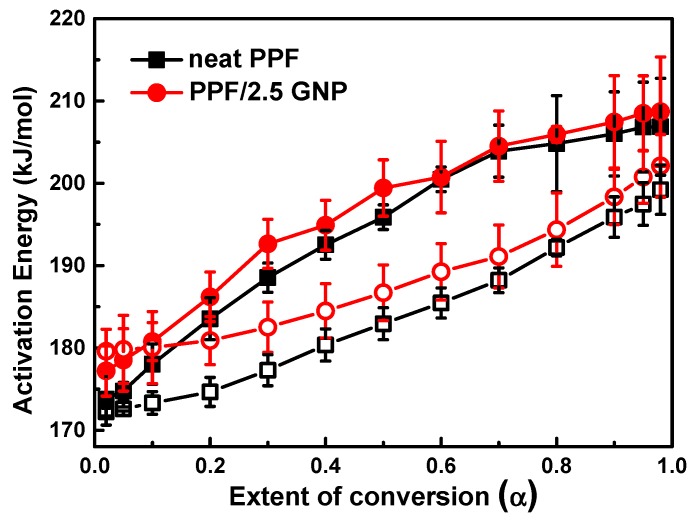
The dependence of activation energy (E) on the extent of conversion (α) for the thermal degradation of neat PPF and PPF/2.5GNP nanocomposite as calculated by Friedman method (filled symbols), and Ozawa Flynn Wall analysis (open symbols).

For the determination of the activation energy by using multiple heating rates, two iso-conversional methods were used: (1) the integral method of Ozawa, Flynn and Wall (OFW) [66], and (2) the isoconversional method of Friedman [67]. The calculated values of activation energy versus the extent of conversion *α* for neat PPF and PPF/2.5 GNP nanocomposite is shown in Figure 5. The differences in the values of E calculated by the OFW and Friedman methods can be explained by a systematic error due to improper integration [68]. According to Figure 5, it seems that the calculated activation energy values of neat PPF and PPF/2.5 GNP nanocomposite slightly increase with increasing the extent of conversion “*α*” presenting less variability among the mean value, especially in the case of the OFW plot. The mean values of neat PPF and PPF/GNP nanocomposites were found to be 193.5 and 195.8 kJ/mol and 183.5 and 188.5 kJ/mol using Friedman and OFW methods, respectively.

Model-fitting was complementary used in the thermal degradation studies of these materials. This method involves fitting different models to α versus temperature curves and simultaneously determining the activation energy *E* and the pre-exponential factor *A*. So, the kinetic model and the parameters for the four heating rates were determined by multivariate non-linear regression method. For this reason, 16 different reaction models were examined through the comparison of the experimental and theoretical data for the conversion range of 0 < *α* < 1. First, it is considered that the degradation of the samples can be described only by one mechanism, without presuming the exact mechanism. If the result of the fitting cannot be considered as accepted, then we must proceed to the fitting of the experimental data with a combination of two mechanisms.

**Figure 6:**
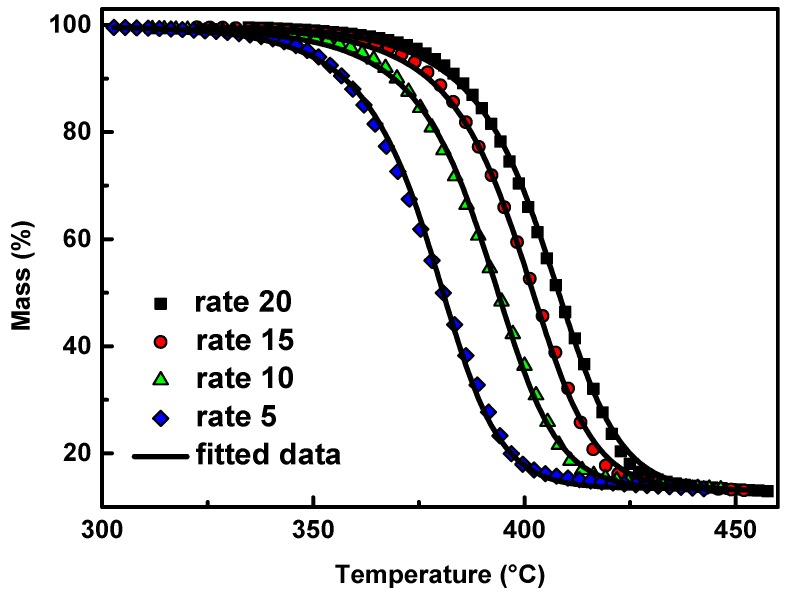
Mass (%) curves of neat PPF at heating rates of 5, 10, 15, and 20 °C/min in a nitrogen atmosphere (symbols) and the corresponding fitting curves with the Cn reaction model (continuous black lines).

The form of the conversion function, given by the best fitting for the neat PPF, is the mechanism of autocatalysis n-order (*Cn*) described by the equation $f\left(\alpha \right)={\left(1-\alpha \right)}^{n}\left(1+K_{cat}X\right)$, where *K_cat_* is the autocatalysis rate constant and *X* the extent of conversion of the autocatalytic reactions. The results of the fitting can be seen for neat PPF (continuous black lines) in Figure 6 with a correlation coefficient of 0.9998; small divergences appear in the final stages of degradation. However, there isn’t any further improvement in the quality of the fitting using two or more reaction mechanisms, since the differences among the regression coefficient values are rather small.

**Figure 7:**
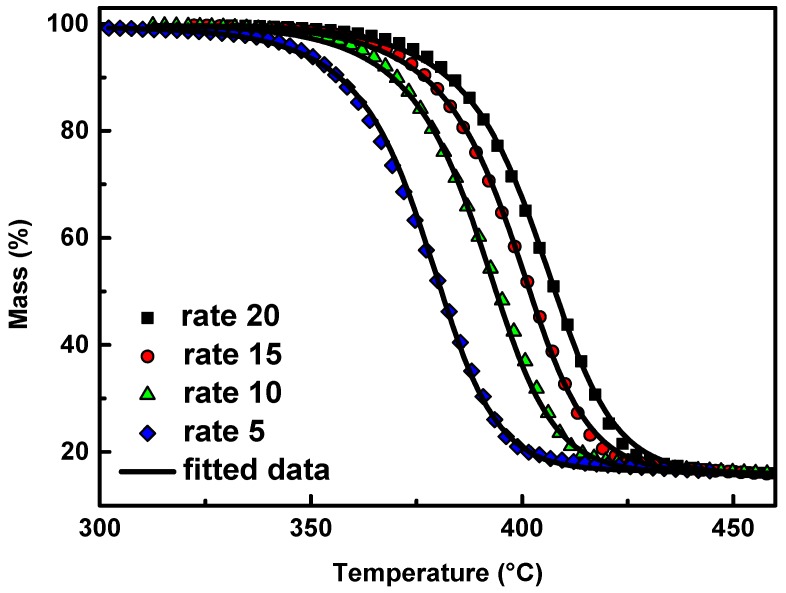
Mass (%) curves of PPF/2.5 GNP nanocomposite at heating rates of 5, 10, 15, and 20 °C/min in a nitrogen atmosphere (symbols) and the corresponding fitting curves with the Cn model (continuous black lines).

Multivariate non-linear regression method also indicated that the *Cn* model, the mechanism of autocatalysis n-order, fit better to thermal decomposition of PPF/2.5 GNP nanocomposite with a correlation coefficient value of 0.9998, Figure 7. This is in consistence with the iso-conversional method (Figure 5) in which the activation energy of materials slightly increases with increasing the extent of conversion, suggesting that a single-step reaction mechanism may efficiently describe the degradation.

The calculated values of the activation energy, pre-exponential factor and the reaction order for PPF and PPF/2.5 GNP nanocomposite using the nth-order with autocatalysis (Cn) model are summarized in Table 2. It was found that the same model describes the reaction mechanism of both neat PPF and PPF/2.5 GNP nanocomposite. It is worthwhile noting that that the activation energy values are close to the ones calculated from OFW analysis, as well as Friedman’s method (Figure 5). Comparing the activation energies, PPF/2.5 GNP nanocomposite has slightly higher values than neat PPF. This improvement in thermal stability of nanocomposite is associated with the 2-dimensional planar structure of GNPs. The mobility of polymer matrix is restricted, and the chemical reactivity of the corresponding chains is lower increasing activation energy and eventually retarding more the thermal degradation of the nanocomposites.

The degradation mechanism was afterwards studied with Py-GC/MS measurements. The compounds are pyrolyzed in a pre-selected temperature and followed by the separation of the evolved pyrolysis products by a GC capillary column and subsequent detection via mass spectroscopy. This method enables the determination of the exact degradation routes that take place when a polymeric material is heated in high temperatures in inert atmosphere.

The degradation mechanisms of furan-based polyesters were first studied by our group, including this of PPF [23,69]. In general, they follow the degradation paths of their terephthalate homologues. Similarly to all polyesters with β-hydrogen atoms on their macromolecular chains, they degrade mainly by heterolytic scission reactions of the hydrogen in β position to the ester bond [70,71,72,73]. This degradation route leads to the evolution of vinyl-ended and carboxyl-ended products. Additionally, homolytic scission reactions of the acyl-oxygen and alkyl-oxygen bonds can occur, especially under higher pyrolysis temperatures [24,31].

**Figure 8:**
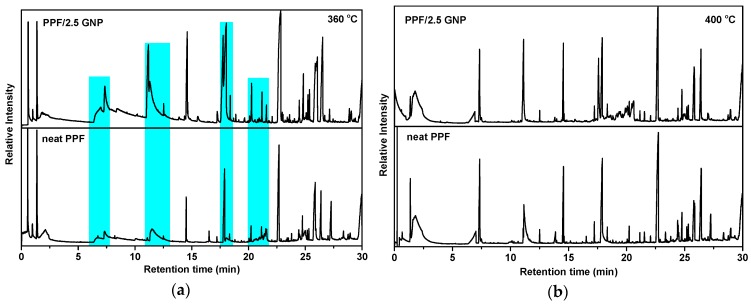
Total Ion Gas chromatographs of neat PPF and PPF/2.5 GNP after pyrolysis at (**a**) 360 °C; (**b**) 400 °C. The highlighted peaks correspond to compounds that are evolved via secondary degradation routes.

**Scheme 2:**
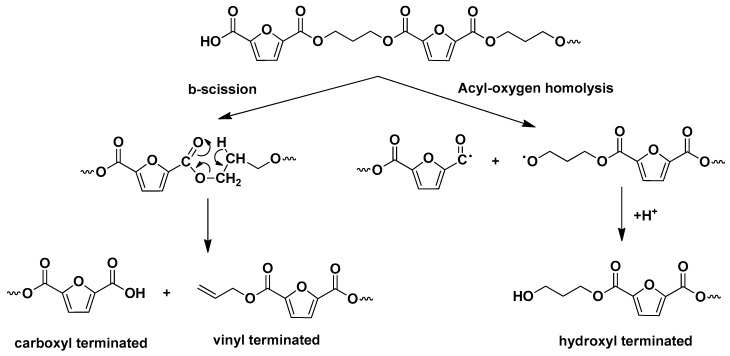
Basic degradation routes of PPF.

Total ion chromatographs of PPF and PPF/2.5 GNP after pyrolysis at 360 °C and 400 °C are presented in Figure 8 and the corresponding compounds, identified via their mass spectra, are presented in Table 3. As expected, the main pyrolysis products are vinyl- and carboxyl- ended molecules that result from β-scission reactions (Scheme 2). Other classes of products are some hydroxyl-ended, methoxy-ended molecules as well as some aldehydes. It should be noted that since the mass spectra of the molecular ions of aldehydes are very weak, we cannot be completely confident about their identification even though they are known to be released from polyesters during heating. All the possible degradation routes have been explained in detail in our previous publications [22,24,25,31,69,74].

In both samples, the pyrolysis products identified were identical, meaning that GNP doesn’t affect the degradation mechanism of PPF. The slight increase in the activation of energy calculated by TGA is caused by the ability of graphene layers to hinder gas diffusion in the polymeric matrix as they can reduce chain mobility [75,76]. When comparing the GC patterns of PPF and PPF/2.5 GNP it is observed that while they don’t have any noteworthy differences at 400 °C, there is a significant increase in the intensity of some peaks, in the presence of GNP at 360 °C suggesting their larger relative amount. Those peaks in Rt = 7.00, 7.35, 11.19, 11.30, 17.76, 20.27, 21.19 and 21.58 min are highlighted in Figure 8a and the majority was identified as compounds that are produced via secondary degradation routes, including -OH ended compounds from acyl-oxygen homolysis and aldehydes from α-hydrogen bond scission. Therefore, the presence of GNP in the PPF matrix resulted in a pronounced occurrence of homolytic degradation reactions in comparison with neat PPF.

## 3. Materials and Methods

### 3.1. Materials

2,5-Furandicarboxylic acid (purum 97%), propylene glycol anhydrous 99.6% (PG) and tetrabutyltitanate (TBT) catalyst of analytical grade were purchased from Sigma-Aldrich (Taufkirchen, Germany). GNPs with the trade name xGnP^®^ - Grade M were supplied by XGSciences (Lansing MI, USA). According to the manufacturer, the nanoplatelets have an average thickness of 6–8 nm, surface area 120–150 m^2^/g and average particle diameter of 5 μm. All other materials and solvents used were of analytical grade and were purchased from Sigma-Aldrich.

### 3.2. Synthesis of 2,5-Dimethylfuran-dicarboxylate (DMFD)

2,5-Furandicarboxylic acid (15.6 g), anhydrous methanol (200 mL) and concentrated sulfuric acid (2 mL) were placed in a round bottom flask (500 mL) and the mixture was refluxed for 5 h. The excess of the methanol was distilled off, and the solution was filtered through a disposable Teflon membrane filter. During filtration, dimethylfurandicarboxylate (DMFD) was precipitated as white powder and, after cooling, distilled water (100 mL) was added. The dispersion was partially neutralized by adding Na_2_CO_3_ 5% *w*/*v* during stirring, while pH was measured continuously. The white powder was filtered and the solid was washed several times with distilled water and dried. The isolated white dimethyl ester was recrystallized with a mixture of 50/50 *v*/*v* methanol/water. After cooling, 2,5-DMFD was precipitated in the form of white needles. The reaction yield was calculated at 75%.

### 3.3. PPF and Nanocomposites Synthesis

PPF was synthesized through the two-stage melt polycondensation (esterification and polycondensation) in a glass batch reactor [21]. DMFD and propylene glycol in a molar ratio of diester/diol = 1/2.2 were charged into the reaction tube of the polyesterification apparatus with 500 ppm of TBT. The reaction mixture was heated at 160 °C under argon flow for 1.5 h, at 170 °C for additional 1.5 h and finally at 180 °C for 2 h. This first step (transesterification) is considered complete after the collection of almost all the theoretical amount of CH_3_OH, which was removed from the reaction mixture by distillation and collected in a graduate cylinder. In the second step of polycondensation, vacuum (5.0 Pa) was applied slowly over a time of about 30 min to remove the excess of diol, to avoid excessive foaming and to minimize oligomer sublimation, which is a potential problem during the melt polycondensation. The temperature was gradually increased (1.5 h) to 220 °C, while stirring speed was also increased to 720 rpm. The reaction continued at this temperature for 1.5 h. Successively, the temperature was increased to 235 °C for 1.5 h and to 250 °C for additional 2 h. PPF-based GNP nanocomposites containing 0.5, 1 and 2.5 wt.% of GNPs were in-situ prepared using also the two-stage melt polycondensation method. Nanofillers were added to the propylene glycol and the dispersion was subjected to sonication for 15 min to obtain a uniform dispersion. Afterwards, the dispersion was added to the reaction tube together with DMFD and TBT catalyst. The reaction continued, as above described for the synthesis of neat PPF. After the polycondensation reaction was completed, neat PPF and PPF/GNP nanocomposites were easily removed, milled and washed with methanol.

### 3.4. Intrinsic Viscosity Measurements

Intrinsic viscosity [*η*] measurements were performed using a Cannon Ubbelohde viscometer (State College, PA, USA) at 30 °C in a mixture of phenol/1,1,2,2-tetrachloroethane (60/40, *w*/*w*). The sample was kept in the above-mentioned mixture at 90 °C until complete dissolution was achieved. The solution was then cooled to room temperature and filtered through a Teflon disposable membrane filter.

### 3.5. Nuclear Magnetic Resonance (NMR)

^1^H-NMR spectra of polyesters were obtained with a Bruker spectrometer (Billerica, MA, USA) operating at a frequency of 400 MHz. A sample concentration equal to 5% *w*/*v* in deuterated trifluoroacetic acid (d-TFA) was used. The number of scans was 10 and the sweep width was 6 kHz.

### 3.6. Wide Angle X-Ray Diffraction Patterns (WAXD)

X-ray diffraction measurements of the samples were performed using a MiniFlex II XRD system from Rigaku Co. (Tokyo, Japan), with CuK_α_ radiation (λ = 0.154 nm) in the angle 2*θ* range from 5° to 60°.

### 3.7. Thermogravimetric Analysis (TGA)

Thermogravimetric analysis of the PPF and PPF/GNP nanocomposites were carried out using a SETARAM SETSYS TG-DTA 16/18 instrument (Caluire, France) by heating the samples from 25 to 600 °C in a 50 mL/min flow of N_2_ at a heating rate of 10 °C/min. For the kinetic analysis study, neat PPF and PPF/GNP nanocomposites with 2.5 wt.% filler content (PPF/2.5 GNP) were heated at four different heating rates, namely 5, 10, 15, and 20 °C/min. Samples (4.5 ± 0.5 mg) were placed in alumina crucibles, while an empty alumina crucible was used as a reference. Continuous recordings of sample temperature, sample weight, first derivative, and heat flow were taken.

### 3.8. Pyrolysis-Gas Chromatography–Mass Spectroscopy (Py-GC/MS)

For Py-GC/MS analysis of polyesters a very small amount of each material is “dropped” initially into the “Double-Shot” EGA/PY-3030D Pyrolyzer (Frontier Laboratories Ltd., Fukushima Japan) using a CGS-1050Ex (Kyoto, Japan) carrier gas selector. For pyrolysis analysis (flash pyrolysis) each sample was placed into the sample cup which afterwards fell free into the Pyrolyzer furnace. The pre-selected pyrolysis temperatures were 360 and 400 °C and the GC oven temperature was heated from 50 to 300 °C at 20 °C/min. Those two temperatures were selected based on the EGA pyrogram and represent the sample prior and after thermal decomposition. Sample vapors generated in the furnace were split (at a ratio of 1/50), a portion moved to the column at a flow rate of 1 mL/min, pressure 53.6 kPa and the remaining portion exited the system via the vent. The pyrolyzates were separated using temperature programmed capillary column of a Shimadzu QP-2010 Ultra Plus (Kyoto, Japan) gas chromatograph and analysed by a Shimadzu MS-QP2010SE mass spectrometer at 70 eV. Ultra ALLOY^®^ metal capillary column from Frontier Laboratories Ltd. (Fukushima, Japan) was used containing 5% diphenyl and 95% dimethylpolysiloxane stationary phase, column length 30 m and column ID 0.25 mm. For the mass spectrometer the following conditions were used: Ion source heater 200 °C, interface temperature 300 °C, vacuum 10^−4^–10^0^ Pa, *m*/*z* range 10–500 amu and scan speed 10.000. The chromatograph and spectra retrieved by each experiment were subjected to further interpretation through Shimadzu and Frontier post-run software (Kyoto, Japan).

## 4. Conclusions

In this work, PPF/GNP nanocomposites containing 0.5, 1 and 2.5 wt.% GNPs were successfully synthesized via the in-situ transesterification and polycondensation method. The addition of GNPs did not affect the intrinsic viscosity, or the chemical structure of the nanocomposites as shown by the ^1^H-NMR spectra and the viscosity measurements. The crystallinity of the as received materials was assessed by WAXD measurements. The diffractograms showed that the materials are amorphous. Thermal properties with focus on thermal stability and degradation mechanism were evaluated. All samples had strong thermal stability, as no remarkable mass loss was observed until 325 °C and have been decomposed in similar one-step procedures as shown in the dTG curves. The study of degradation kinetics through thermogravimetry revealed a small increase in the activation energy value for the nanocomposite with 2.5 wt.% GNP in comparison with neat PPF. The nanofillers did not alter the degradation pathways of PPF, as both the β-scission and the acyl-oxygen homolysis occur. Instead, they affected the balance between the primary heterolytic scission reactions and the homolytic degradation routes. The presence of GNP in the PPF matrix resulted in a pronounced occurrence of homolytic degradation reactions in comparison with neat PPF as more -OH ended compounds were detected in the chromatographs.

## Figures and Tables

**Table 1:** TGA results of all studied samples.

Sample	T_d,max_ (°C)
Neat PPF	393.2
PPF/0.5 GNP	394.2
PPF/1 GNP	395.9
PPF/2.5 GNP	396.3

**Table 2:** Activation energy, pre-exponential factor and reaction order of neat PPF and PPF/2.5 GNP nanocomposite.

Sample	Mechanism	Activation Energy/kJmol^−1^	Pre-exponential Factor/s^−1^	Reaction Order/n	Log K_cat_	Regression Coefficient
Neat PPF	Cn	187.0	12.2	1.36	0.81	0.9998
PPF/2.5 GNP	Cn	191.1	12.6	1.43	0.82	0.9998

**Table 3:** Possible pyrolysis products of neat PPF and PPF/2.5 GNP nanocomposite.

PPF		PPF/2.5 GNP		Mw (amu)	Possible Product
Rt (min)		Rt (min)			
360	400	360	400		
-	0.24			44	CO_2_ or acetaldehyde
0.55	-	0.59		76	1,3-propanediol
1.36	1.36	1.37	1.37	44	CO_2_ or acetaldehyde
-	1.8	1.79	1.78	68	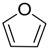
6.74	7.02	7.00	6.93	112	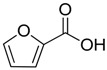
7.33	7.35	7.35	7.34	152	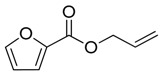
11.09	11.09	11.19	11.13	162	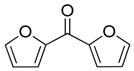
11.51	11.16	11.30	-	170	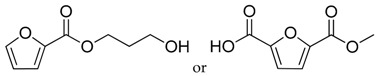
12.50	12.51	12.53	12.51	190	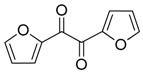
-	13.88	13.88	13.82	196	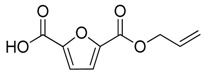
14.52	14.57	14.61	14.56	236	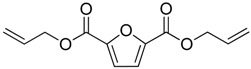
-	14.62	14.65	14.62	238	
-	-	17.76	17.58	254	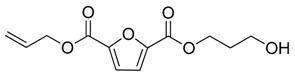
17.89	17.91	18.05	17.90	264	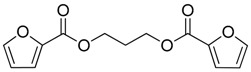
20.22	-	20.27	20.23	322	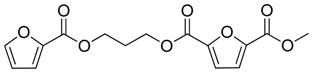
21.14	21.15	21.19	21.15	256	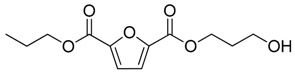
21.52	21.54	21.58	21.54	336	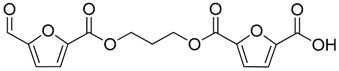
22.69	22.73	22.83	22.70	348	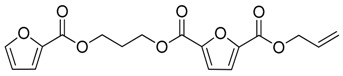
24.42	24.50	24.47	24.43	388	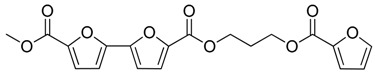
24.75	24.77	24.84	24.76	358	unidentified
25.18	25.19	25.23	25.19	366	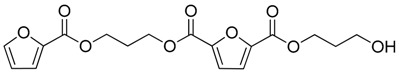
25.31	25.32	25.38	25.32	350	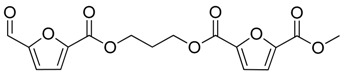
25.88	25.85	25.88	25.81	392	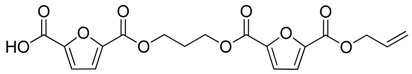
26.38	26.43	26.53	26.41	432	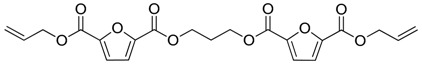
27.28	27.25	27.14	27.00	434